# Levothyroxine treatment for congenital hypothyroidism based on thyroid function: a 10-year clinical retrospective study

**DOI:** 10.1186/s12902-022-01061-z

**Published:** 2022-05-28

**Authors:** Shan He, Xiaolin Ma, Jinghui Yang, Li Li

**Affiliations:** Department of Pediatrics, The First People’s Hospital of Yunnan Province, NO. 157, Jin Bi Road, Kunming, 650032 Yunnan China

**Keywords:** Congenital hypothyroidism, Levothyroxine, Thyroid stimulating hormone, Endocrine, Children

## Abstract

**Objective:**

To explore the appropriate dosage of levothyroxine treatment for congenital hypothyroidism patients with different thyroid stimulating hormone (TSH) levels.

**Methods:**

A total of 116 patients, who were regularly followed-up in our endocrine clinic from January 2010 to December 2020, were divided into four groups based on their thyroid function (group A: TSH ≥ 100 mIU/L, group B: TSH ≥ 20, and < 100, group C: TSH > 4.6 mIU/L and < 20 mIU/L with free thyroxine (FT4) < 6.6 pmol/L, and group D: TSH > 4.6 mIU/L and < 20 mIU/L with FT4 > 6.6 pmol/L). The initial dosage of levothyroxine was individualized for each patient based on their TSH level and then adjusted according to their thyroid function at every follow-up time point. The levothyroxine dosage at each time point was compared between the groups, and thyroid function and physical and neurological development after treatment were also compared.

**Results:**

After individualized dosage adjustment, all patients achieved normal thyroid function. Although there were statistical differences in neurological development between the four groups (*p* < 0.05), development was within the normal range for all groups.

**Conclusion:**

An individualized levothyroxine dosage can provide the same therapeutic effect compared to the recommended dosage. This strategy may also reduce the risk of a drug overdose.

## Background

Congenital hypothyroidism (CH) is a common endocrine disease with an incidence of 1:2000 to 1:4000 in newborns; it is one of the most common preventable causes of intellectual disability worldwide [1]. Although oral levothyroxine is the first choice for CH treatment, the side effects associated with levothyroxine overdoses, such as hyperactivity, lethargy, tachycardia, tachypnea, dyspnea, abnormal pupillary light reflexes, vomiting, and diarrhea, cannot be neglected. Neonates with markedly elevated thyroid stimulating hormone (TSH) levels (> 40 mIU/L) in newborn screening should be treated with levothyroxine immediately; confirmative testing is not needed. Neonates with a TSH level of 20 mIU/L to 40 mIU/L, or 6 mIU/L to 20 mIU/L and with a low free thyroxine (FT4 < 6.6 pmol/L) level, should also be treated with levothyroxine [2]. However, the intervention remains controversial for infants with a mildly elevated TSH level (within 6 mIU/L to 10 mIU/L) and a normal FT4 level. To date, there are no widely recognized guidelines for precise levothyroxine dosage based on CH severity, and research regarding levothyroxine dosage adjustment during the therapeutic phase is also rare. The present study aimed to explore the effects of variations in levothyroxine dosage in patients with different initial THS levels over a course of treatment to help guide CH management in clinical practice.

## Materials and methods

### Study design, population, and data collection

**Fig. 1:**
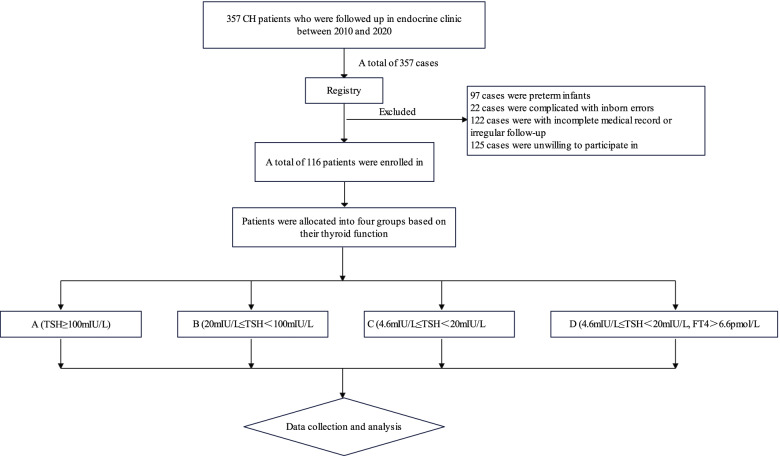
Study design flowchart

Patients who were regularly followed-up in our endocrine clinic between January 2010 and December 2020 were enrolled in this study. All legal guardians were informed and signed the informed consent. This study was approved by the research ethics committee of The First People’s Hospital of Yunnan Province. The inclusion criteria were as follows: (1) patients who were diagnosed with CH based on the *Consensus statement on the diagnosis and management of congenital hypothyroidism* issued by the Subspecialty Group of Endocrinologic, Hereditary and Metabolic Diseases, The Society of Pediatrics, Chinese Medical Association [3], (2) a gestational age between 37 and 42 weeks, (3) having complete medical records, and (4) regular follow-up. The exclusion criteria were as follows: (1) preterm infants, (2) incomplete medical records, (3) irregular follow-up, (4) refusal to participate in this study, and (5) being complicated with other inborn errors. A total of 116 patients were enrolled in this study. Of the 241 patients excluded, 97 were preterm infants, 22 were complicated with inborn errors, and 122 had incomplete medical records or irregular follow-up. In addition, 125 patients were unwilling to participate in our study but had similar baseline characteristics to enrolled patients. A flow chart of the study design is shown in Fig. 1.

### Patient groups

Patients were categorized into four groups based on their thyroid function at the time of diagnosis. The patient groups included group A: TSH level > 100 mIU/L, group B: TSH ≥ 20 and < 100, group C: TSH > 4.6 mIU/L and < 20 mIU/L with free thyroxine (FT4) < 6.6 pmol/L, and group D: TSH > 4.6 mIU/L and < 20 mIU/L with FT4 > 6.6 pmol/L (defined as subclinical hypothyroidism, whose TSH persisted at an elevated level after two consecutive rechecks).

### Institutional regime of levothyroxine replacement therapy in our unit

Group A patients, whose TSH level was ≥100 mIU/L, received 10 μg/kg·d as the initial levothyroxine dosage, which was at the lower limit of the recommended dosage. Group B patients, who had a moderately elevated TSH level (20 mIU/L ≤ TSH < 100 mIU/L), were administered a dosage of 5–8 μg/kg·d. Groups C and D, who had a mildly elevated TSH level (4.6 mIU/L < TSH < 20 mIU/L), were administered 3–4 μg/kg·d levothyroxine. However, as the specification of levothyroxine is 50 μg per tablet, for dividing convenience, it is usually prescribed as “1 ½ Tab or 1 2/3 Tab … .”

### Data collection

Maternal thyroid function was tested through antenatal care, and the clinicians would inquire about the results once their babies were confirmed. The initial levothyroxine dosage information for the four groups was collected. TSH levels of the four groups were analyzed 1 month after treatment to determine if they had reached the normal range. In addition, physical and neurological development were evaluated and compared between groups at the age of 1, 2, and 3 years.

### Thyroid function test

Blood samples of patients were collected, and serum was isolated and transferred to our laboratory for thyroid function tests. A time-resolved fluorometry kit (Wallac, Finland) was adopted for the TSH test following the manufacturer’s instructions; while T4, T3, FT4, and FT3 were tested using a time-resolved immunofluorescence assay (TRFIA) kit (Xin Bo Biotech, China) and analyzed using an immunofluorescence analyzer (EFFICUTA, China), strictly following the manufacturer’s instructions.

### Physical and neurological development evaluation

Growth monitoring was conducted by qualified clinicians at the age of 1, 2, and 3 years; height, weight, and head circumference were also documented. The Gesell Development Scale score was adopted for neurological development assessment and performed at the age of 1, 2, and 3 years, including evaluating gross and fine motor development, adaptability, and sociability.

### Statistical analysis

Continuous variables with normal distribution were expressed as mean ± standard deviation (SD) and assessed by t-test, whereas skewed-distribution variables were summarized as medians and interquartile ranges and assessed using the Mann-Whitney U test. Categorical variables were demonstrated by frequencies and proportions (%) and analyzed by Pearson’s chi-square test or Fisher’s exact test. SPSS 26.0 software (Chicago, IL, USA) was employed for statistical processing, and a two-tailed *P*-value < 0.05 was considered statistically significant.

## Results

### General characteristics of patients

There were no significant differences in sex distribution, birth weight, height at birth, head circumference at birth, or cesarean section delivery rate among the four groups (*p* > 0.05). However, the differences in abnormal maternal thyroid function rate (maternal hypothyroidism or subclinical hypothyroidism) and the initial TSH screening levels among the four groups were significant (Table 1).

**Table 1:** General characteristics of patients

	Group A(*n* = 37)	Group B(*n* = 26)	Group C (*n* = 17)	Group D(*n* = 36)	*P*-value
Gender					
male (%)	18(48.6)	14(53.8)	8(47.1)	24(37.5)	0.387^*^
Cesarean section (%)	9(24.3)	4(15.4)	6(35.3)	8(22.2)	0.507^*^
Abnormal maternal thyroid function (%).”	14(37.8)	2(7.7)	3(17.6)	3(8.3)	0.004^*^
Birth weight					
mean ± SD (kg)	3.1 ± 0.23	3.0 ± 0.23	3.2 ± 0.27	3.0 ± 0.28	0.058^$^
Height at birth					
mean ± SD (cm)	50.1 ± 1.04	50.0 ± 0.77	49.8 ± 1.36	50.0 ± 1.14	0.830^$^
Head circumference at birth					
mean ± SD (cm)	34.2 ± 0.65	34.0 ± 0.54	33.9 ± 0.71	33.8 ± 0.85	0.269^&^
Initial TSH screening (mIU/L)					
median (range)	100 (15.5333)	23.4(8.5420)	11(4,9)	15(4237)	0.000^&^

^*^Fisher’s exact test

^$^One-way ANOVA

^&^Kruskal-Wallis test

### Diagnostic time and thyroid function of patients

All patients were diagnosed with CH at around 1 month of life; there was no significant difference in the diagnostic time point between the 4 groups. The TSH and FT4 levels of the four groups of patients at diagnosis are presented in Table 2.

**Table 2:** Age and TSH and FT4 levels of patients at diagnosis

	Group A(*n* = 37)	Group B(*n* = 26)	Group C(*n* = 17)	Group D(*n* = 36)	F	*P*
Age (days)	30(20, 40)	30(20, 37)	30(30, 40)	30(22.5, 50)	1.8902	0.5955^*^
TSH (mIU/L) median (range)	100(100, 111.94)	49.845(30.49, 78.36)	11.8(9.82, 16.51)	10.6935(7.88, 14.81)	100.5532	< 0.0001^*^
FT4 (pmol/L) median (range)	4.01(2.08, 5.09)	6.585(4.657, 9.15)	5.5(5, 5.91)	10.83(9.585, 14.3)	72.5549	< 0.0001^*^

*Kruskal-Wallis test

### Dosage of levothyroxine at each follow-up point

After being given an initial dose of thyroxine based on their TSH level, some patients in each group still did not exhibit normal thyroid function at 1 month. Then the levothyroxine dosage for each patient was adjusted at each time point (Table 3).

**Table 3:** Levothyroxine dosages at different time points for the four patient groups (μg/kg. d)

Time	Group A	Group B	Group C	Group D	F	*P*
At diagnosis	9.12 ± 2.43	7.9 ± 2.19	6.28 ± 2.40	4.47 ± 2.03	28.17	< 0.0001
2 weeks	8.76 ± 2.68	7.6 ± 2.18	5.19 ± 2.03	4.24 ± 1.93	21.77	< 0.0001
1 month	7.35(5.81,9.15)	6.96(6.1,8.33)	4.72(4.72,4.72)	3.3(2.47,4.91)	51.4976	< 0.0001
3 months	5.7(3.99,7.43)	5.15(3.85,5.77)	3.11(3.11,3.11)	2.86(2.08,3.39)	50.793	< 0.0001
6 months	4.14(3.11,4.9)	3.43(2.94,4.17)	2.67(2.67,2.67)	2.17(1.43,3.01)	40.3017	<.0001
12 months	3.2(2.53,3.89)	2.5(2.31,2.87)	2.21(2.21,2.21)	1.69(1.1,2.5)	38.6064	<.0001
18 months	2.9(2.2,3.57)	2.23(1.64,2.46)	2.07(2.07,2.07)	1.6(1.19,2.12)	28.1057	<.0001
24-month	2.95 ± 0.92	2.27 ± 1	1.97 ± 0.8	1.58 ± 0.51	10.43	<.0001
36-month	2.5(2.24,3.6)	1.68(1.12,2.99)	1.69(1.69,1.69)	1.22(1.08,1.8)	15.4036	0.0015

### Thyroid function restoration in each group

**Fig. 2:**
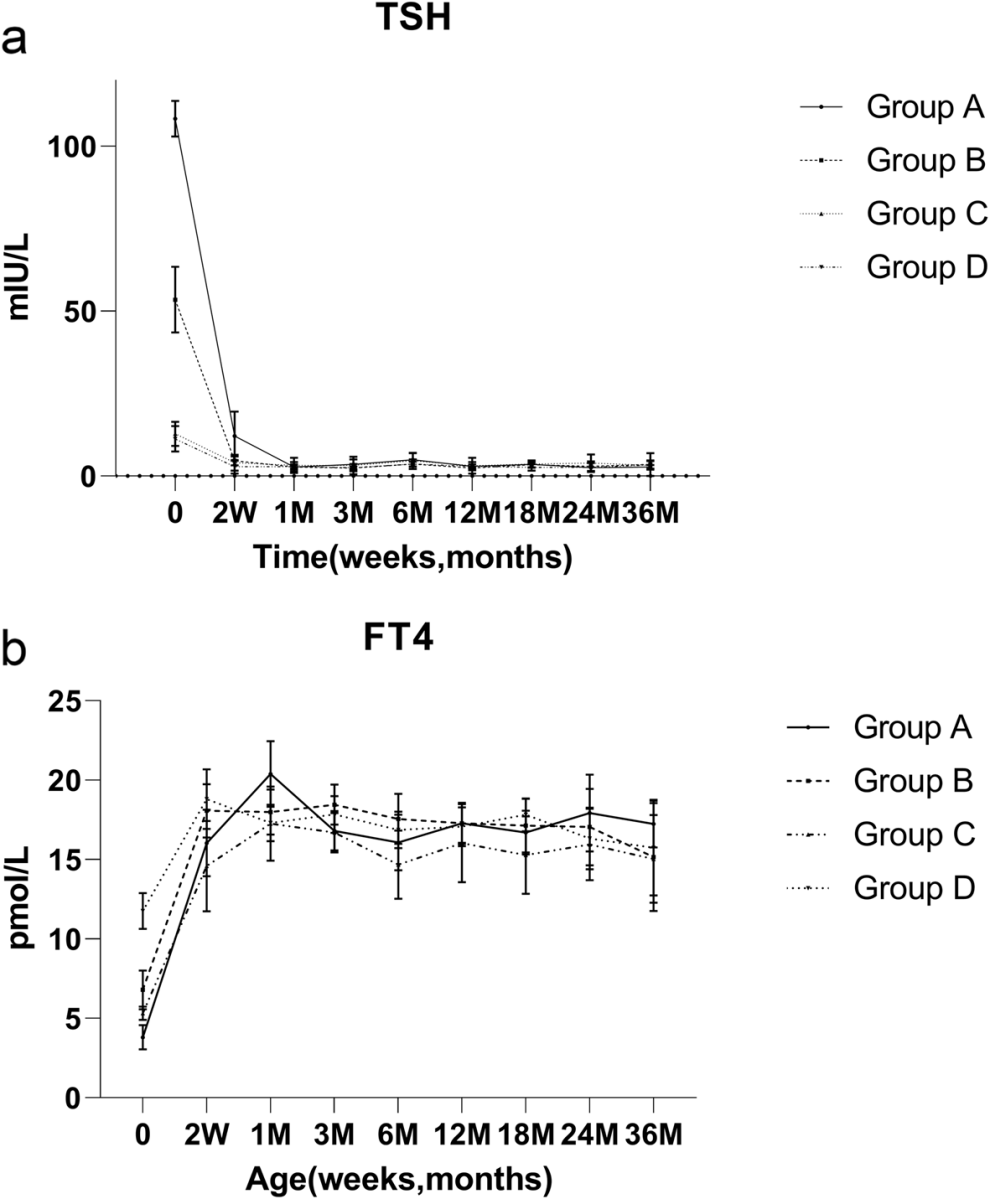
Thyroid function restoration in each group. **a** Changes in thyroid stimulating hormone (TSH) level of patients after treatment. **b** Changes in free thyroxine (FT4) level of patients after treatment

Two weeks after the intervention, the TSH level of group A was still higher than that of the other three groups; however, at the following time points, there was no difference between the four groups (*p* > 0.05) (Fig. 2a). The FT4 concentrations of the four groups showed no significant difference after treatment (*p* > 0.05); all levels gradually increased and then stabilized at the normal range at the follow-up time points (Fig. 2b).

### Physical and neurological development of the four groups after treatment

After treatment, there was no difference in height, weight, or head circumference among the four groups (*p* > 0.05). While a statistical difference in neurological development among the four groups was observed (*p* < 0.05), the Gesell Developmental Scores were all within the normal range (Table 4).

**Table 4:** Gesell developmental score at different ages for the four patient groups

	Group A	Group B	Group C	Group D	F	*P*
**1 year**						
Gross motor	89.65 ± 6	90.5 ± 6.85	93.06 ± 5.83	91.42 ± 5.6	1.37	0.2552**
Fine motor	91.08 ± 8.29	93.15 ± 6.69	96.35 ± 5.89	94.42 ± 5.77	2.71	0.0485**
Adaptability	91(86,97)	93(88,98)	96(96,96)	90(89,97)	4.6743	0.1973*
Language	88(82,90)	86(80,90)	95(95,95)	90(87,94)	14.6561	0.0021*
Sociability	90(88,98)	95(88,98)	98(98,98)	92.5(89,98)	8.9311	0.0302*
**2 years**						
Gross motor	94(90,99)	96(91,98)	92(92,92)	96.5(90,101)	2.7147	0.4377*
Fine motor	94(88,98)	93(89,98)	92(92,92)	97.5(93,98)	5.443	0.1421*
Adaptability	95(89,99)	90.5(87,99)	93(93,93)	99(95,102)	7.9004	0.0481*
Language	84(79,89)	85.5(83,92)	96(96,96)	93(90,96)	24.4284	< 0.0001*
Sociability	97.5(89.5101)	94(89,99)	98(98,98)	98.5(92,102)	1.196	0.7540*
**3 years**						
Gross motor	97.5(89.5101)	94(89,99)	98(98,98)	98.5(92,102)	10.6981	0.0135*
Fine motor	91.42 ± 8.7	96.5 ± 4.22	96.86 ± 5.71	97.76 ± 5.01	3.8	0.0146**
Adaptability	90(88,94)	97.5(92,99)	95.5(95.5,95.5)	98(93,100)	15.2357	0.0016*
Language	90(88,97)	93(90,93)	95(95,95)	93(90,98)	2.2074	0.5305*
Sociability	93.38 ± 7.19	97.45 ± 5.85	95.93 ± 5.44	95.82 ± 4.53	1.38	0.2576**

*Kruskal-Wallis test

**One-way ANOVA

## Discussion

Congenital hypothyroidism is one of the most common preventable causes of intellectual disability worldwide, and the first choice of treatment is oral levothyroxine. Thyroid hormones play a critical role in brain and somatic development, specifically in children under 2 years of age when their neurological development is highly dependent on thyroid hormones [4]. Research has shown that thyroid hormones are critical factors in the formation and differentiation of neurons that must be constantly available. Therefore, many clinical guidelines recommend using a high initial dosage of levothyroxine (10–15 μg/kg·d), regardless of the cause and severity of CH, to ensure a serum FT4 (or T4) concentration in the upper half of the pediatric reference range and a serum TSH level in the normal range for age, is achieved as soon as possible [5–7]. However, a few studies have shown that using a lower thyroxine dosage than recommended could also achieve the same goal while reducing the risk of thyroxine overdose [5, 8]. Excessive serum FT4 levels may lead to craniosynostosis (premature fusion of one or more cranial sutures), developmental-behavioral impairment, and attention deficit hyperactivity [8–10]. Moreover, levothyroxine might also have a negative effect on intelligence quotients during puberty [11, 12].

In our retrospective observation of patients administered individualized dosages of levothyroxine according to their initial TSH level at diagnosis, after 1 month, there were four patients (10.8%) in group A, one patient (3.8%) in group B, four patients (23.5%) in group C, and three patients (8.3%) in group D who did not reach the normal TSH level. Furthermore, there were eight patients (21.6%) in group A, one patient (3.8%) in group B, and one patient (2.7%) in group D who had an FT4 level beyond the upper limit of normal values at 1 month following treatment. At the follow-up time, the dosage of levothyroxine for each patient was adjusted according to thyroid function, and their thyroid function eventually returned to a normal range. Our study suggests that to reduce the risk of levothyroxine overdose, individualized levothyroxine dosage should be adjusted. Currently, there is no exact dosage for patients with different TSH levels; however, for most patients with mildly elevated TSH, low-dose thyroxine could restore their TSH level to normal 1 month after treatment. It is critical to follow up regularly during treatment to monitor thyroid function and adjust the thyroxine dosage to maintain thyroid function within the normal range.

Research performed by Soliman et al. (2012) revealed that 25% of the patients (*n* = 45) treated with a high dose of levothyroxine (10–15 μg/kg·d) developed hyperthyroidism [13]. Craven and Frank (2018) reported that a high initial levothyroxine dosage (> 12.5 μg/kg·d) may have caused hyperthyroidism. After a period of follow-up, more than half of the patients required a reduction in their dosage; thus, the authors suggested reducing the initial dosage to avoid overtreatment [14]. Nevertheless, comparative studies between individualized treatment and high initial dosage treatment are scarce. We explored the correlation between levothyroxine dosage and patient thyroid function and between thyroid function restoration time and patient TSH levels after individualized treatment through a clinical retrospective study.

An identical high initial dose of levothyroxine for all CH patients has been challenged by Mathai et al., who retrospectively explored the strategy of using a variable initial dosage of levothyroxine. They categorized CH patients by etiology and administered levothyroxine at dosages of 10, 12, and 15 μg/kg·d for patients who were diagnosed with thyroid hormone synthesis disorder, ectopic thyroid, and thyroid agenesis, respectively. After treatment, they successfully normalized the serum FT4 of all patients within 14 d. Furthermore, the authors also demonstrated that a lower initial thyroxine dosage (9.98 ± 3.19 μg/kg·d) could also cause FT4 levels to return to normal within 16 d [15]. Another study performed by Bakker et al. (2002) was carried out in 30 newborns with CH who received levothyroxine dosage ranging from 4.8–11.1 μg/kg·d. They found that there was no association between the initial dosage and the time of FT4 normalization, either in the low-dose group (6.4 ± 2.1 μg/kg·d) or the high-dose group (11.8 ± 1.4 μg/kg·d); both groups reached normal FT4 and TSH levels in a similar time-frame [16]. Tuhan et al. (2016) used three different dosages (6–9.9 μg/kg·d, 10–11.9 μg/kg·d, and 12–17 μg/kg·d) for CH treatment and showed that there was no difference in TSH levels at 1 month after treatment [17].

The patients enrolled in our study were diagnosed at around 1 month of life; this delay was due to regional factors and cultural disparities. Our hospital’s province is a remote and backward area in China, and resources are limited. After the initial screening was positive, many parents were notified via text message. However, many family members did not pay much attention to this message because of their limited medical knowledge, and their babies were usually asymptomatic. Thus, the parents and patients did not return for follow-up in time. Chinese women have the custom of confinement and are reluctant to go outside, unless necessary, during confinement.

All patients were treated within 2 months of birth in the present study. The levothyroxine dosage was adjusted to maintain their thyroid function at each follow-up, which remained within the normal range. There was no difference in physical and neurological development between the treated groups (*p* > 0.05); however, we could not determine whether these results were due to the small sample size. Therefore, further prospective, multicenter control studies are required. Although there was a statistical difference in the Gesell Development Scale score between the groups (*p* < 0.05), they were all within the normal range. As reported in the literature, if CH patients are treated within 1 month of birth, they can gain a normal IQ; the longer hypothyroidism goes undetected and untreated, the lower the IQ achieved [6, 18–20].

In our study, even for patients with significantly elevated TSH levels, the initial individualized dosage of levothyroxine received was still lower than recommended; yet, they eventually achieved normal thyroid function with no physical or neurological developmental impairment. In summary, the key factor in the successful treatment of CH is early detection and intervention, rather than a high levothyroxine dosage.

## Limitations

The present study has limitations, including that it was retrospective and the sample size was small, which may lead to unreliable conclusions. Furthermore, we only enrolled term infants, whereas the prevalence of CH is higher in preterm infants than in term neonates. Thus, our study was not universally representative. Given the importance of precise levothyroxine treatment, further prospective studies are required.

## Conclusion

Compared to the recommended dosage, an individualized levothyroxine dosage can provide the same therapeutic effect. And such strategy might reduce the risk of a drug overdose.

## Data Availability

The original data sets and materials are not publicly available because of patient privacy protection but are available from the corresponding author upon reasonable request.

## Abbreviations

TSH: Thyroid-stimulating hormone
FT4: Free thyroxine
CH: Congenital hypothyroidism
IQ: Intelligence quotient
T3: Triiodothyronine
T4: Thyroxine
